# Unusual Case of Fatal Suffocation Due to Balloon Aspiration in a 23-Month-Old Infant

**DOI:** 10.7759/cureus.32398

**Published:** 2022-12-11

**Authors:** Mohammed Y ALnami, Taher M Sumayli, Jalal A Hakami

**Affiliations:** 1 Pediatric Emergency Medicine, King Fahad General Hospital, Jazan, SAU; 2 Pediatric Intensive Care, King Fahad General Hospital, Jazan, SAU; 3 Pediatrics Emergency Medicine, King Fahad General Hospital, Jazan, SAU

**Keywords:** pediatrics emergency, respiratory distress, foreign body aspiration, cyanosis, balloon

## Abstract

Foreign body aspiration is a serious medical emergency and the fifth leading cause of accidental injury-related fatalities. This case involves a 23-month-old infant who aspirated two latex balloons and developed cardiac arrest during balloon extraction. He was revived, but a CT scan revealed hypoxic encephalopathy.

## Introduction

Foreign bodies in the upper respiratory tract are a significant source of illness and death at both ends of the age spectrum. Tracheobronchial foreign body aspirations are a common cause of acute respiratory distress, persistent lung damage, and even mortality in young infants [1]. It is a life-threatening condition with the highest morbidity in children younger than three years old. It is also the leading cause of unintentional infant mortality [2].

Hazelnuts, pistachio nuts, sweets, beans, popcorn, grapes, dried grapes, carrots, sausages, and plant seeds are common foreign bodies. Typical aspirated foreign material consists of pen caps, coins, balloons, marbles, tiny fragments of different toys, and pins. Balloon aspiration in children is lethal [3]. It is the leading cause of choking fatalities in children. More children are killed by balloons than by any other type of foreign body [4]. Although infants had the highest mortality rate, 25% of the deaths involved youngsters aged six years or older [5]. We present a fatal balloon aspiration in a 23-month-old infant who developed hypoxic encephalopathy after a cardiac arrest.

## Case presentation

**Figure 1:**
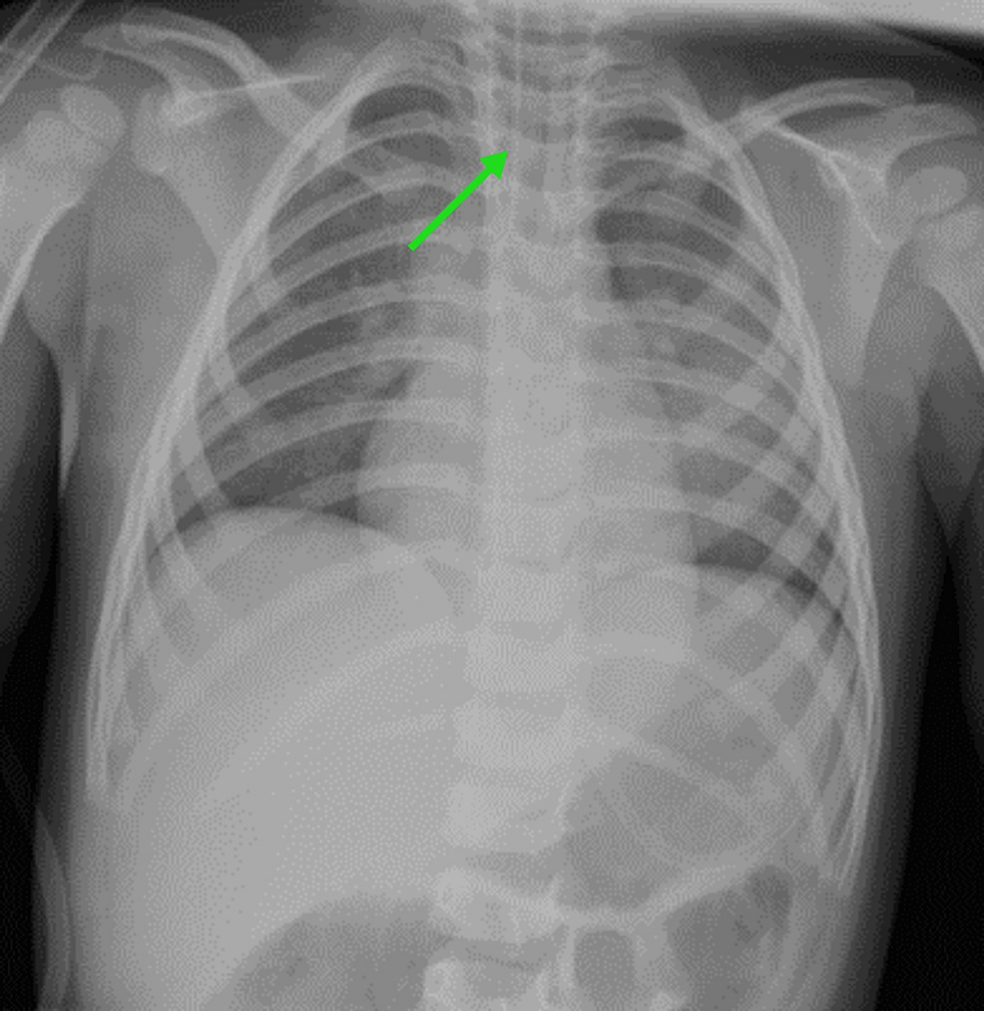
X-ray chest showing the tube in place and a clear lung field

A 23-month-old girl was brought to the emergency department after aspirating two balloons. One was extracted intact immediately at home. The child became restless, coughed, became drowsy and cyanosed, and was rushed to a nearby hospital, where she began gasping, developed cardiorespiratory arrest, and was resuscitated for 15 minutes. She was intubated there, and while intubation was underway, another balloon was retrieved from the laryngeal inlet by the intensive care unit (ICU) doctor. She was intubated successfully and transferred to our hospital for further management. On examination, the child was intubated, her vital signs were stable, and some body movements were observed, like the flickering of the eyes, tongue, and lip movement. The patient was given fentanyl at a rate of 2 micrograms per kilogram per hour, and the ENT department was consulted, who cleared the patient from their side. An X-ray showed a tube in place, and both lungs were clear (Figure 1).

Lung auscultation revealed good air entry with equal bilateral. Her laboratory investigations are shown in Table 1.

**Table 1:** Baseline investigations of the patient during the course of illness WBC = white blood count; BUN = blood urea nitrogen; INR = international normalized ratio

Lab workup	1^st^ day	2^nd^ day	3^rd^ day	4^th^ day	5^th^ day
Hemoglobin (mg/dl)	13.6	10.1	9.7	10.8	-
WBC (10^9^/L)	9.97	13.32	8.91	12.37	-
Platelets (10^9^/L)	505	238	268	249	-
Sodium (mmol/L)	145	159	172	173	157
Potassium (mmol/L)	3.7	3.02	3.4	5.87	5.78
BUN (mg/dl)	5.8	3.1	1.9	0.5	2.5
Creatinine (mg/dl)	45	33	18	51	41
Magnesium (mmol/L)	1.01	0.78	0.81	0.72	0.75
Calcium (mmol/L)	2.18	2.22	2.07	1.86	2.04
INR	1.21	1.79	2	2.4	1.54
ALT (U/L)	-	25.8	-	-	-
Protein (g/L)	-	37.3	-	-	-
Albumin (g/L)	-	25.9	-	-	-

**Figure 2:**
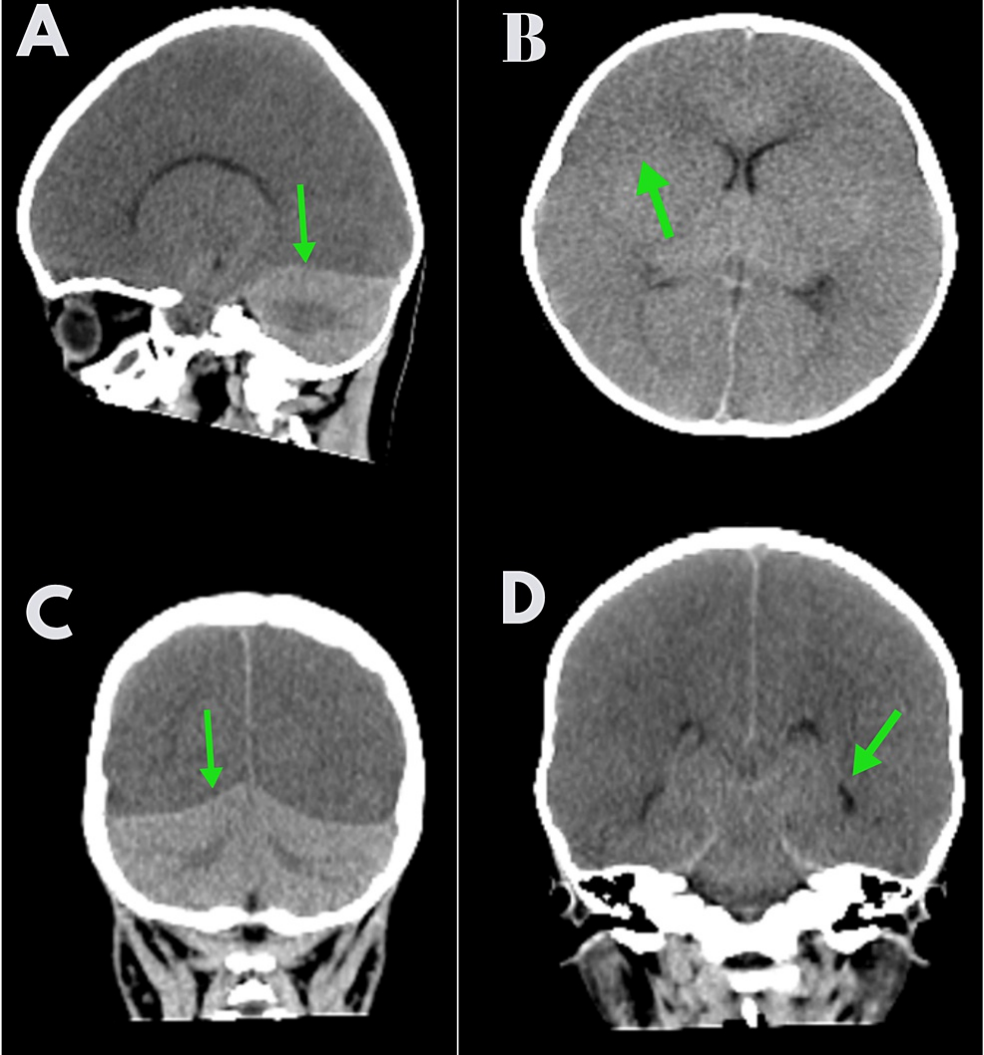
CT head showing diffuse brain edema with hypoxic-ischemic encephalopathy A: Sagittal view showing a generalized decrease in the density of the supratentorial brain due to edema with a relatively normal cerebellum, which appears as a white cerebellum sign; B: Axial view showing diffuse brain edema with loss of gray matter differentiation in cerebral hemispheres; C & D: Coronal reformatted image showing white cerebellum sign and effacement of the basal cisterns.

She was started on clindamycin and cefotaxime. She was on ventilator support, with synchronized intermittent mandatory ventilation (SIMV) with a fraction of inspired oxygen (FiO2) of 40%, positive end-expiratory pressure (PEEP) of 5, ventilation rate (VR) of 30, and PC of 12; her saturation remained above 94%. On the ventilator, she developed abnormal movements, generalized tonic-clonic rhythm, tachycardia, and high blood pressure. She was started on midazolam at 1 microgram per kilogram per minute for a presumed seizure. In the evening, the patient developed status convulsion, and she was loaded with phenytoin at 10 mg/kg and later sedated with midazolam (18 mcg/kg/min), fentanyl (2 mcg/kg/hr), phenytoin (8 mg/kg/day), and phenobarbitone (6 mg/kg/day). Her clinical finding revealed bilaterally fixed and dilated pupils, no reaction, and no cough or gag reflex. Computed tomography (CT) head was done and showed diffuse brain edema with hypoxic-ischemic encephalopathy (Figure 2).

She was diagnosed with a case of severe brain asphyxiation secondary to prolonged arrest after foreign body inhalation. The family was counseled regarding the prognosis and outcomes as the patient was deteriorating every day. On the fifth day of admission, the patient went into cardiopulmonary arrest and died.

## Discussion

Children, particularly those under three years, are susceptible to foreign body aspiration due to their propensity to put anything in their mouths. Due to the lack of molars, young children chew their food incompletely using their incisors. In addition, toddlers who are sucking or blowing up balloons are at risk of asphyxia [6].

Multiple studies indicate that males are more likely than girls to experience foreign body aspiration, and the majority of patients are younger than three years old [7]. The size, shape, and consistency of foreign items, as well as the location of the obstruction, appear to be significant predictors of fatal obstruction. Furthermore, knowing the mechanisms of accidental inhalation of these compounds may aid in the development of legislation and efficient preventive measures [8]. The five cases of choking on latex balloons were analyzed to highlight the significance of the consistency of inhaled bodies [4,9]. This clearly shows that after balloons have entered the airway, they are less likely to be ejected, as pliable items do not readily disassemble, which would assist their ejection.

Complications of foreign body inhalation are classified as minor and major. Williams et al. classified complications as minor, including arterial oxygen desaturation, bradycardia, and bronchospasm [10]. Laryngeal edema, pneumothorax, and cardiac arrests are major outcomes of foreign body aspiration. Hypoxic ischemic brain damage is the key factor of survival following cardiac arrest. It is linked with neurologic impairments ranging from minor cognitive abnormalities to limited consciousness and prolonged vegetative states [11]. Preventing the development of significant effects requires an early diagnosis and appropriate treatment [12].

The treatment plan is contingent on several variables, including the patient's overall state, the clinical environment, and the health facility's policy requirements. Treatment of a balloon aspiration can be quite challenging. The Heimlich technique, the standard therapy for choking, may be unsuccessful. Parents may attempt to remove the foreign body themselves, perhaps delaying the implementation of effective management. Attempting to remove the balloon by wiping the back of the child's throat with a finger might be hazardous, as it will just push the balloon farther down and cause more complications. While the foregoing methods are being evaluated in the hospital, an emergency bronchoscopy should be prepared. Flexible bronchoscopes are generally utilized for diagnostic purposes. However, current research has established a new function for them in the treatment of foreign bodies [12].

## Conclusions

Most foreign body aspiration is preventable. The aspiration of a balloon is an extremely dangerous and difficult foreign body in the larynx. Such aspiration can be prevented by educating parents and the general public properly. The case can help establish a new technique for foreign body aspiration removal and increase cultural awareness regarding the severity of balloons.
